# Quantification of the response of circulating epithelial cells to neodadjuvant treatment for breast cancer: a new tool for therapy monitoring

**DOI:** 10.1186/bcr1328

**Published:** 2005-10-04

**Authors:** Katharina Pachmann, Oumar Camara, Andreas Kavallaris, Uwe Schneider, Stefanie Schünemann, Klaus Höffken

**Affiliations:** 1 Klinik für Innere Medizin II der Friedrich Schiller-Universität Jena, Jena, Germany; 2 Transfusionsmedizinisches Zentrum Bayreuth, Bayreuth, Germany; 3 Frauenklinik, Friedrich Schiller-Universität Jena, Jena, Germany

## Abstract

**Introduction:**

In adjuvant treatment for breast cancer there is no tool available with which to measure the efficacy of the therapy. In contrast, in neoadjuvant therapy reduction in tumour size is used as an indicator of the sensitivity of tumour cells to the agents applied. If circulating epithelial (tumour) cells can be shown to react to therapy in the same way as the primary tumour, then this response may be exploited to monitor the effect of therapy in the adjuvant setting.

**Method:**

We used MAINTRAC^® ^analysis to monitor the reduction in circulating epithelial cells during the first three to four cycles of neoadjuvant therapy in 30 breast cancer patients.

**Results:**

MAINTRAC^® ^analysis revealed a patient-specific response. Comparison of this response with the decline in size of the primary tumour showed that the reduction in number of circulating epithelial cells accurately predicted final tumour reduction at surgery if the entire neoadjuvant regimen consisted of chemotherapy. However, the response of the circulating tumour cells was unable to predict the response to additional antibody therapy.

**Conclusion:**

The response of circulating epithelial cells faithfully reflects the response of the whole tumour to adjuvant therapy, indicating that these cells may be considered part of the tumour and can be used for therapy monitoring.

## Introduction

One of the major obstacles to improving breast cancer treatment is the lack of a sensitive and specific assay with which to evaluate the effect of therapy in the adjuvant setting and in metastatic disease. It has been shown that cells can be shed from the tumour at all stages of disease and that these cells may remain in the patient's circulation for lengthy periods after initial treatment of the primary tumour [1,2], but in a proportion of patients these can eventually develop into metastases. Numbers of such cells vary depending on the method and sample, from very few if bone marrow mononuclear cells are analyzed (median 2 per 2 × 10^6 ^in breast cancer patients) [3] to between 5 and 20,000 cells per 7.5 ml blood sample (corresponding to 6 to 25,000 cells per 2 × 10^6 ^mononuclear blood cells) [4] in metastatic breast cancer. Using magnetic bead enrichment and microfluorimetry [5] in lung cancer patients after surgery we had identified numbers of cells [6] similar to those reported by Cristofanilli and coworkers [4]. Recently, we improved this method to avoid cell loss resulting from enrichment procedures and centrifugation, and we were able to detect even higher numbers of epithelial cells in patients with malignant epithelial tumours (between 50/ml and 300,000/ml in more than 90% of patients) [7]. The question then arises regarding whether these epithelial cells, which are detectable in such high numbers, are indeed tumour cells. Preoperative chemotherapy in breast cancer patients [8,9] provides a model in which to address this question. In these patients the initial size of the tumour, as analyzed by magnetic resonance imaging before therapy, can be compared with the size determined by pathological analysis of the remaining tumour tissue after therapy at surgery [10]. This reduction in size can be correlated to the reduction in numbers of blood epithelial cells.

## Materials and methods

In order to measure the reduction in circulating epithelial cells induced by neoadjuvant chemotherapy, we monitored epithelial cells before each therapy cycle in 30 patients with breast cancer treated with two different neoadjuvant chemotherapy schedules [11,12]. Only the first three (dose intensified) epirubicin or four epirubicin/cyclophosphamide cycles of the regimen were considered in the analysis because measurement of the numbers of cells during the following cycles was confounded by release of cells from disintegrating tumour tissue (Camara O and coworkers, unpublished data).

**Figure 1:**
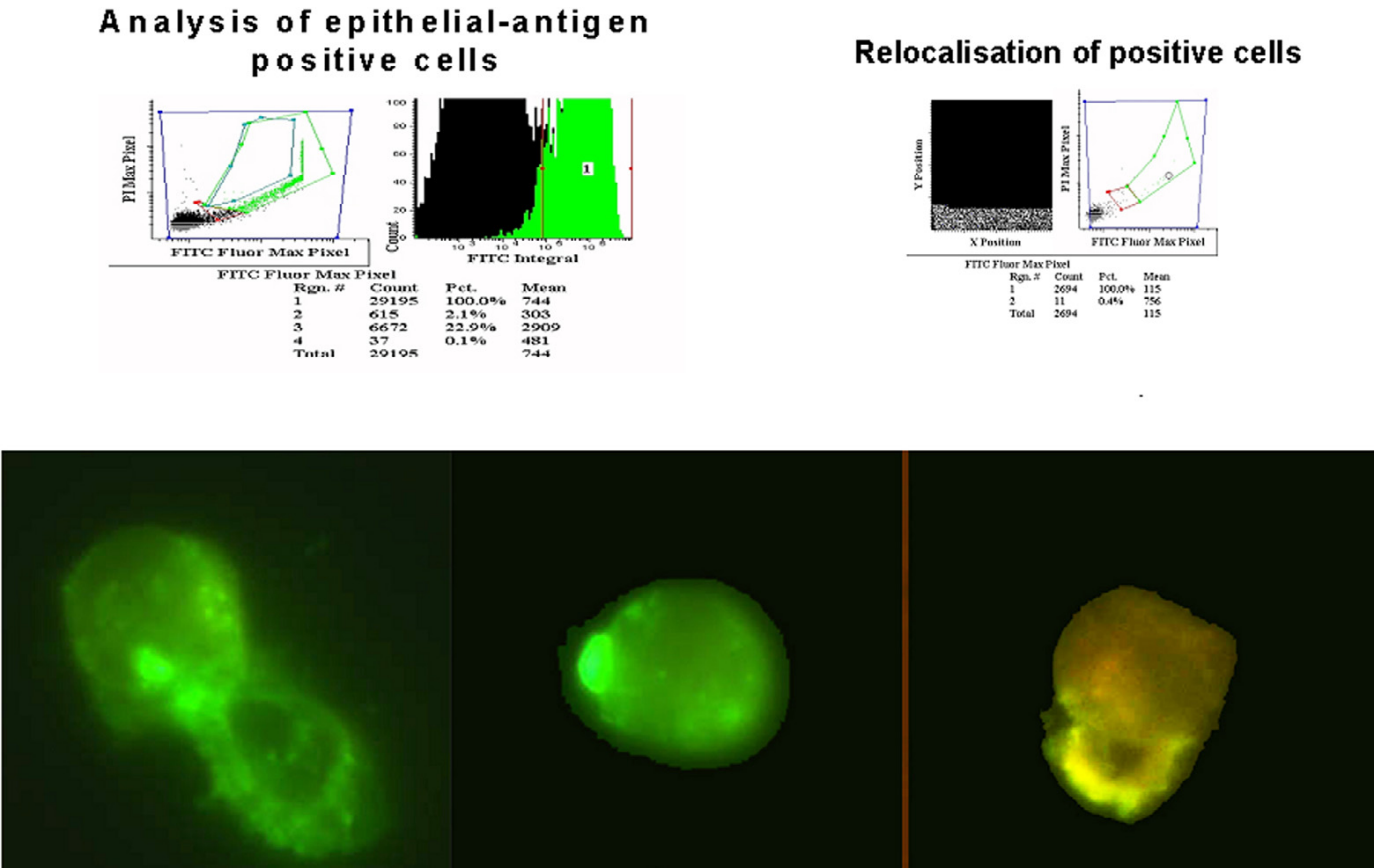
Fluorimetric analysis, relocalization and fluoromicrograph of individual circulating tumour cells. Shown are the procedure for quantification and visual control (1 hour for cell preparation and analysis) and three typical viable circulating tumour cells (green fluorescing cap). One cell is also stained for oestrogen receptor (orange fluorescence).

Once informed consent had been obtained from all participants, as required for ethics committee approval, peripheral blood anticoagulated with EDTA was drawn before the start of therapy and before each of the following therapy cycles. As a control, blood from 25 patients with nonepithelial haematological malignancies in different stages of disease (seven with chronic myelogenous leukaemia (five in clinical remission and two who had relapsed), five with acute lymphocytic leukemia (two in clinical remission), five with acute myelogenous leukemia (two in clinical remission after allogeneic stem cell transplantation) and eight patients with plasmocytoma (five in clinical remission after autologous stem cell transplantation and three who had relapsed)) was also analyzed for circulating cell staining with the antiepithelial antibody. First, red blood cells were lysed using ammonium chloride, which was followed by a single centrifugation step. Then, the pellet of white cells was collected (in accordance with a previously described approach [7]) and incubated with FITC-conjugated mouse anti-human epithelial antibody (HEA; Miltenyi Bergisch Gladbach, Germany), and live cells in suspension were applied to a poly-L-lysine treated slide, which was analyzed using a Laser Scanning Cytometer^® ^(Compucyte Corporation, Cambridge, MA, USA) [11]. This method, termed MAINTRAC^® ^analysis, enables relocation of cells for visual examination and quantification, and for taking fluoromicrographs. Typical cells (green fluorescence, exclusively located at the surface, indicative for viability, often forming caps) are shown in Fig. 1; note that the right-most cell is counter-stained for the oestrogen receptor.

## Results

In previous experiments [7] we performed combination staining with anti-CD45-PE and restaining with cytokeratin, and found that CD45^+ ^blood cells could easily be discriminated from epithelial cells, and that all cells staining with anti-EpCAM (epithelial cell specific adhesion molecule) also stained with cytokeratin. Nonspecific staining with anti-EpCAM can occur when one is using fixed cells and intracellular staining, but this was not a problem when live cells were analyzed, which were defined as cells exhibiting exclusive surface staining. In the previous study [7] no live epithelial antigen positive cells were detected in 97% of healthy donors aged between 17 and 75 years.

**Figure 2:**
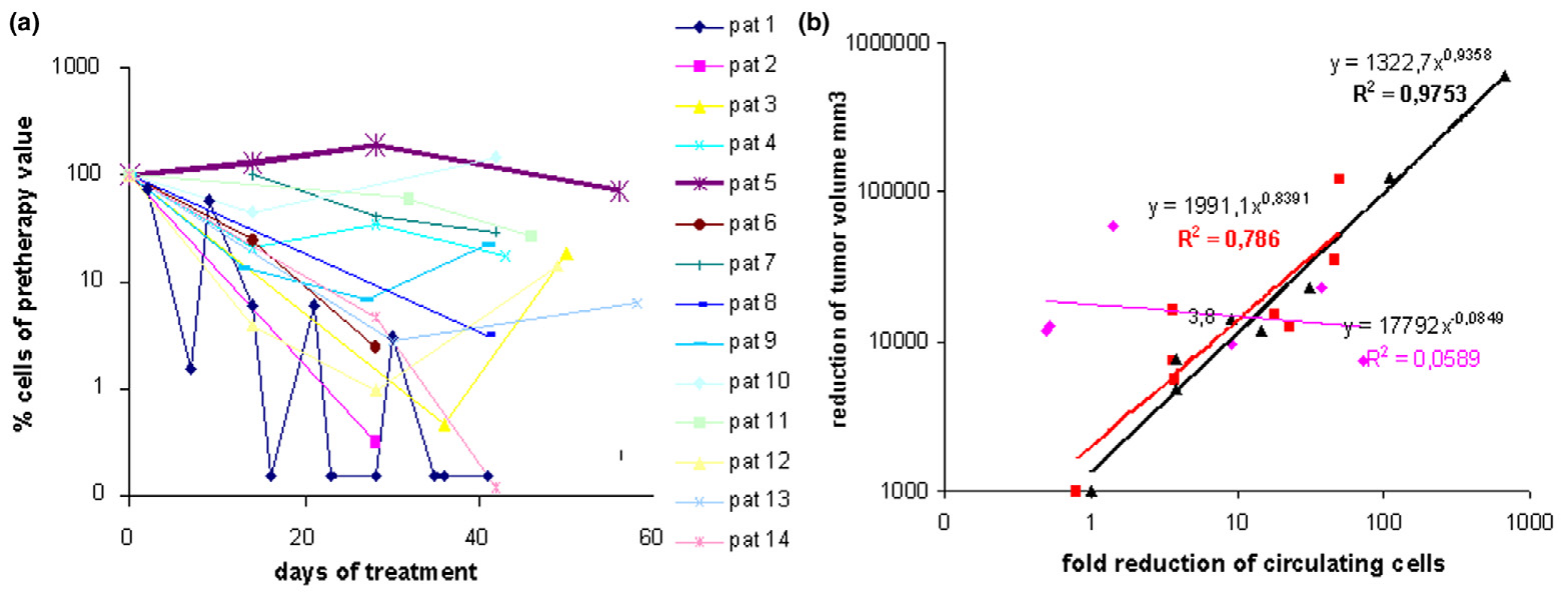
Changes in circulating tumour cell numbers. **(a) **Fourteen typical courses of changes in cell numbers in breast cancer patients monitored during neoadjuvant (primary) therapy. **(b) **Comparison of the response of circulating tumour cells during the first three (or four) therapy cycles versus tumour reduction in the therapy regimen without herceptin (thick lines) and the regimen including herceptin (thin line).

In none of the patients with haematological malignancies, whether full blown, in complete remission with regenerating haematopoiesis, or in relapse, could we detect cells staining with the HEA antibody. In breast cancer patients assigned to neoadjuvant treatment, live epithelial cells were detected in all patients before the start of therapy, with pretherapy numbers between 600 and 273,150 cells/ml (mean 11,876 cells/ml). Duplicate analyses differed by less than 10%. Typical longitudinal analyses (normalized to pretherapy values, which were set at 100%) during the three epirubicin cycles are shown for 14 patients in Fig. 2a. The decrease in the number of circulating tumour cells (CTCs) from different breast cancer patients varied, and the difference was up to several hundred-fold, indicating that cells from individual patients respond differently to the same therapy schedule. The same was true for the 16 patients treated with the epirubicin/cyclophosphamid schedule (not shown).

Reductions in cell numbers (the nadir of circulating cell numbers was used) were strongly correlated (R^2 ^= 0.97) with final tumour reduction in the patients receiving dose-intensified epirubicin (calculated as the difference between pretherapy volume (determined using magnetic resonance) and remaining tumour volume at surgery (determined by the pathologist); 8/14 patients underwent surgery; Fig. 2b and Table 1).

The epirubicin/cyclophosphamide group was further divided into patients who did not receive subsequent herceptin therapy (7/8 underwent surgery) and HER2/neu-positive patients who received herceptin treatment after chemotherapy (6/8 underwent surgery; Table 1). The correlation coefficients between CTC reduction with initial chemotherapy and tumour volume reduction was R^2 ^= 0.79 in the chemotherapy-only group and R^2 ^= 0.059 in the group in which chemotherapy was followed by herceptin treatment. It should be noted that measurements were performed during epirubicin/cyclophosphamide cycles only; this is because later analyses may be perturbed by release of cells from decaying tumour tissue and therefore were not included. Thus, although the circulating epithelial cells in the HER2/neu-positive group did not respond adequately to epirubicin/cyclophosphamide, tumours frequently exhibited a good response at the end of therapy after application of herceptin. The lack of correlation in the herceptin treatment group indicates that subsequent antibody treatment contributes to tumour reduction in a different manner, which is not reflected in the initial response of CTCs to chemotherapy.

## Discussion

In patients with breast cancer we found that the first few courses of neoadjuvant chemotherapy had identical influence on CTC numbers, and that therapy reduced CTCs in the same way as it affected the tumour *per se*. Because circulating epithelial cells detected using our approach respond to chemotherapy in the same way as the tumour does, it is likely that these cells in peripheral blood of untreated breast cancer patients stem from the tumour. Also, the strong correlation between the reduction in CTCs with chemotherapy and the final reduction in size of the tumour indicates that monitoring of CTCs, even during the first cycles of therapy, predicts whether the tumour ultimately will respond adequately to this therapy [13] (the course of which lasts almost half a year). Unnecessary toxicity during neoadjuvant therapy could therefore be avoided by discontinuing ineffective therapy.

In contrast, in the HER2/neu-positive group circulating epithelial cells did not respond adequately to chemotherapy, but a large proportion of the tumours in ultimately exhibited complete response to the entire therapy (including chemotherapy and antibody therapy). This is in good agreement with the known reduced responsiveness of HER2/neu-positive tumour cells to chemotherapy (other than with subsequent antibody therapy [14], during which analyses were not performed). Importantly, such analyses conducted during adjuvant treatment, for which no other marker is yet available, could – for the first time – help in determining immediately whether the applied therapy is effective.

The significance of circulating epithelial cells in tumour patients is still unclear. It is not known how long such cells survive [2] or to what extent they are able to form metastases. It appears that the potential to grow into metastases may be restricted to a small fraction [15] of 'stem cells' [16]. Therefore, it may not be the simple number of CTCs but rather their behaviour (decrease or increase) during the course of disease that may predict patient outcome.

## Conclusion

The method presented here permits easy, rapid, reliable and reproducible repeated quantification of epithelial cells in peripheral blood, and could serve as a tool for real-time monitoring of therapy *in vivo*. This will become especially valuable in the adjuvant setting, where therapy has until now been given without any ability to measure the efficacy of treatment.

## Abbreviations

CTC = circulating tumour cell.

## Competing interests

The authors declare that they have no competing interests.

## Authors' contributions

KP conceived of the study, was responsible for its design and coordination and drafting and redrafting of the manuscript, OC recruited the patients and made major contributions to interpreting the results, AK and US were responsible for treatment of the patients, providing the blood samples, SSch performed the measurements and data collection and KH supervised the study and critically read the manuscript. All authors read and approved the final manuscript

## Figures and Tables

**Table 1:** Correlation between reduction in tumour size and reduction in circulating tumour cells with treatment

Patient group	Tumour size before treatment (mm)	Tumour size at surgery (mm)	Fold reduction in circulating cell numbers
Patients receiving dose-dense epirubicin	25 × 25 × 25	12 × 12 × 12	9.09
	25 × 20 × 25	9 × 12 × 8 + small remnant tumour structures	14.70
	70 × 50 × 25	70 × 50 × 20	1.00
	85 × 85 × 85	38 × 26 × 22	666.67
	50 × 50 × 50	0	111.11
	60 × 60 × 60	ND	16.00
	35 × 35 × 20	11 × 11 × 11	31.00
	25 × 25 × 25	20 × 20 × 20	3.80

Patients receving epirubicin/cyclophosphamide without herceptin	22 × 15 × 28	13 × 10 × 8	3.70
	50 × 50 × 50	15 × 10 × 12	50.00
	25 × 25 × 25 + DCIS	7 × 7 × 7 + small remnant tumour structures	18.18
	40 × 30 × 30	27 × 27 × 27	3.68
	36 × 40 × 26	15 × 15	46.00
	50 × 30	25 × 35 × 17	0.80
	15 × 11 × 13 and 8 × 9 × 8	17 × 15 × 9	23.00

Patients receiving epirubicin/cyclophosphamide with herceptin	80 × 70 × 70	Not yet available	
	50 × 30 × 40	0	1.40
	20 × 32 × 20	5 × 5 × 5	0.53
	38 × 26 × 37	Right: 26 × 17; left: 17 × 13 and 16 × 9 and 7 × 6 and 3 × 5	37.60
	20 × 24 × 20	0	9.20
	32 × 18 × 20	0	0.50
	20 × 15 × 20	0	71.67

Tumour size (as determined using magnetic resonance) before initiation of treatment and tumour size at surgery (as determined by the pathologist) and reduction in circulating epithelial cells. Patients received dose-dense epirubicin (three cycles), epirubicin/cyclophosphamide (four cycles) without subsequent herceptin treatment, or epirubicin/cyclophosphamide (four cycles) and subsequent herceptin. DCIS, ductal carcinoma *in situ*; ND, not determined.

## References

[B1] Meng S, Tripathy D, Frenkel EP, Shete S, Naftalis EZ, Huth JF, Beitsch PD, Leitch M, Hoover S, Euhus D (2004). Circulating tumor cells in patients with breast cancer dormancy. Clin Cancer Res.

[B2] Pachmann K (2005). Long-time recirculating tumor cells in breast cancer patients. Clin Cancer Res.

[B3] Schindlbeck C, Janni W, Shabani N, Rack B, Gerber B, Schmitt M, Harbeck N, Sommer H, Braun S, Friese K (2004). Comparative analysis between the HER2 status in primary breast cancer tissue and the detection of isolated tumor cells in the bone marrow. Breast Cancer Res Treat.

[B4] Cristofanilli M, Hayes DF, Budd GT, Ellis MJ, Stopeck A, Reuben JM, Doyle GV, Matera J, Allard WJ, Miller MC (2005). Circulating tumor cells: a novel prognostic factor for newly diagnosed metastatic breast cancer. J Clin Oncol.

[B5] Pachmann K, Heiss P, Demel U, Tilz G (2001). Detection and quantification of small numbers of circulating tumour cells in peripheral blood using laser scanning cytometer (LSC). Clin Chem Lab Med.

[B6] Rolle A, Gunzel R, Pachmann U, Willen B, Hoffken K, Pachmann K (2005). Increase in number of circulating disseminated epithelial cells after surgery for non-small cell lung cancer monitored by MAINTRAC^® ^is a predictor for relapse: a preliminary report. World J Surg Oncol.

[B7] Pachmann K, Clement JH, Schneider CP, Willen B, Camara O, Pachmann U, Hoeffken K (2005). Standardized quantification of circulating peripheral tumor cells from lung and breast cancer. Clin Chem Lab Med.

[B8] Cance WG, Carey LA, Calvo BF, Sartor C, Sawyer L, Moore DT, Rosenman J, Ollila DW, Graham M (2002). Long-term outcome of neoadjuvant therapy for locally advanced breast carcinoma: effective clinical downstaging allows breast preservation and predicts outstanding local control and survival. Ann Surg.

[B9] Mamounas EP, Fisher B (2001). Preoperative (neoadjuvant) chemotherapy in patients with breast cancer. Semin Oncol.

[B10] Gajdos C, Tartter PI, Estabrook A, Gistrak MA, Jaffer S, Bleiweiss IJ (2002). Relationship of clinical and pathologic response to neoadjuvant chemotherapy and outcome of locally advanced breast cancer. J Surg Oncol.

[B11] Ruhl I, Bauerfeind I, Kahlert S, Untch M, Hepp H (2004). Neoadjuvant therapy of breast cancer. Gynakol Geburtshilfliche Rundsch.

[B12] Smith IC, Miller ID (2001). Issues involved in research into the neoadjuvant treatment of breast cancer. Anticancer Drugs.

[B13] Schwartz GF, Hortobagyi GN (2004). Proceedings of the consensus conference on neoadjuvant chemotherapy in carcinoma of the breast, April 26–28, 2003, Philadelphia, Pennsylvania. Cancer.

[B14] Wenzel C, Hussian D, Bartsch R, Pluschnig U, Locker GJ, Rudas M, Gnant MF, Jakesz R, Zielinkski CC, Steger GG (2004). Preoperative therapy with epidoxorubicin and docetaxel plus trastuzumab in patients with primary breast cancer: a pilot study. J Cancer Res Clin Oncol.

[B15] Naumov GN, Townson JL, MacDonald IC, Wilson SM, Bramwell VH, Groom AC, Chambers AF (2003). Ineffectiveness of doxorubicin treatment on solitary dormant mammary carcinoma cells or late-developing metastases. Breast Cancer Res Treat.

[B16] Dontu G, Wicha MS (2005). Survival of mammary stem cells in suspension culture: implications for stem cell biology and neoplasia. J Mammary Gland Biol Neoplasia.

